# Sister Mary Joseph's nodule: Three case reports

**DOI:** 10.1186/1757-1626-1-182

**Published:** 2008-09-24

**Authors:** Andreas Larentzakis, Dimitrios Theodorou, Klio Fili, Anna Manataki, Vasiliki Bizimi, Michael Tibishrani, Stylianos Katsaragakis

**Affiliations:** 1 1st Department of Propaedeutic Surgery, Hippokrateion General Hospital, Athens Medical School, University of Athens, Q. Sofias 114 av., 11527, Athens, Greece; 2 Department of Radiology, 'Thriassio' General Hospital, G. Gennimata av., 19600, Attiki, Greece

## Abstract

**Background:**

An umbilical metastatic lesion is called 'Sister Mary Joseph's nodule'. It is an uncommon clinical or radiographic finding, and it is rare as the first sign of a malignant disease.

**Case presentation:**

We report three cases of Sister Mary Joseph's nodule. In the three cases presented, the primary tumor was an adenocarcinona of the sigmoid colon, a carcinoma of the bladder, and an adenocarcinoma of the gallbladder, respectively.

**Conclusion:**

The differential diagnosis of an umbilical lesion should always include metastatic disease apart from benign lesions and primary neoplasms.

## Background

An umbilical lesion can be either benign or malignant. A malignant umbilical mass can represent a primary or metastatic lesion. The term 'Sister Mary Joseph's nodule' was first used by Sir Hamilton Bailey, in 1949 [1], in order to describe the entity of metastatic umbilical lesions. We present three cases of Sister Mary Joseph's nodule.

## Cases presentation

### Case 1

**Figure 1:**
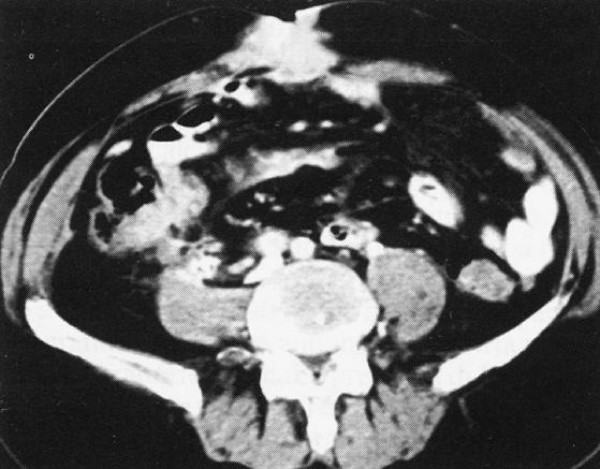
CT-scan of the abdomen: This image shows an umbilical mass measured 3 cm, with indefinite margins.

A 62-year old man presented with a growing periumbilical mass and a mild lower abdominal pain during the last two months. He had symptoms of fatigue, and loss of weight and constipation alternating with diarrhea during the last six months. Patient was receiving medication for hypertension. On clinical examination there was a slight tenderness over the lower abdomen and an umbilical lesion fixed to the underlying tissues. Further evaluation, revealed anemia (Hct: 30%). Colonoscopy detected a tumor of the sigmoid colon. Computed tomography (Figure 1) showed a mass measured 3 cm, with characteristics of non-homogeneous soft structures, located to the anterior abdominal wall, at the region of the umbilicus, involving the adjacent portion of rectus abdominis muscle and adipose tissue. Both biopsies of umbilical and sigmoid tumors revealed the presence of adenocarcinoma. Considering the advanced stage of the disease, patient received chemotherapy as primary treatment.

### Case 2

**Figure 2:**
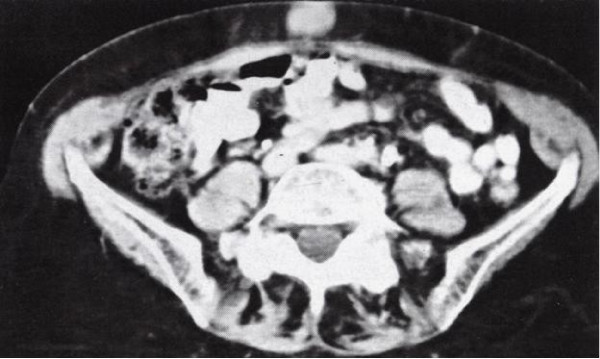
CT-scan of the abdomen: This image shows an oval umbilical mass measured 2 cm.

A 64-year old female presented with haematuria and dysuria during the last 4 months. She had no other medical or surgical history. The assessment with ultrasound detected an established left hydronephrosis and a mass at the left lateral wall of the bladder protruding into its cavity. Cysteoscopic biopsy of the mass showed a transitional cell carcinoma. Further assessment with computed tomography (CT-scan) confirmed the presence of the tumor at the left lateral wall of the bladder, and revealed pelvic lymph nodes involvement and an umbilical mass measured 2 cm (Figure 2). Percutaneous biopsy of the umbilical mass also showed transitional cell carcinoma. Patient underwent cystectomy and chemotherapy.

### Case 3

**Figure 3:**
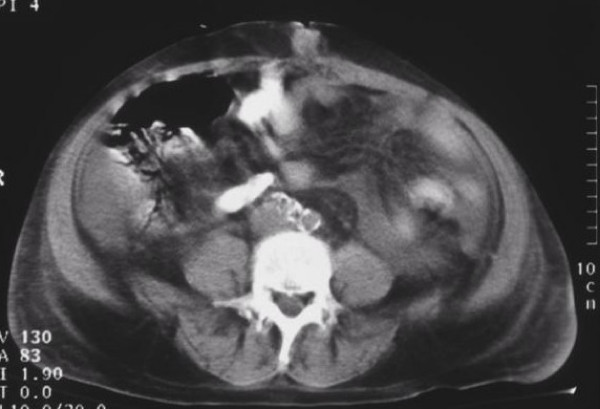
CT-scan of the abdomen: This image shows an umbilical mass measured 2.5 cm, involving the adjacent adipose tissue.

A 54-year old male presented with an umbilical painless mass. He was receiving medication for diabetes mellitus. Patient's physical examination revealed no other abnormality. Pathologic examination of percutaneous biopsy specimen showed malignant epithelial tumor, with characteristics of gall bladder or pancreatic origin adenocarcinoma. Neither upper gastrointestinal endoscopy nor colonoscopy detected any abnormality. Computed tomography of the abdomen showed a gall bladder with increased wall thickness and intraluminal invasive mass, peritoneal lesions, ascites, and an umbilical mass involving the adjacent adipose tissue (Figure 3). Patient received surgical treatment. The pathologic examination of the surgical specimen showed adenocarcinoma of the gallbladder. Patient's further treatment was chemotherapy.

## Discussion

The occurrence of Sister Mary Joseph's nodule is uncommon, and as first sign of malignancy is rare [2]. The intestinal and genitourinary tracts represent the most common, but not the only ones, primary malignant sites [3]. However, the study of an umbilical mass as unique clinical finding should be directed by the suspicion of being a metastatic deposit [4], having also in mind the potential of a primary malignant umbilical lesion or a benign disease [5]. In addition, an umbilical mass in a patient with known malignancy, especially of the abdomen, should be evaluated as potential spreading of the primary disease, a fact that can influence the therapeutic decision making [4,6]. Finally, the approach of surgery should be under careful consideration in these cases, because Sister Mary Joseph's nodule represents the spreading of a neoplasm, which is accompanied by low survival rates [4].

## Conclusion

An umbilical mass can represent a benign lesion, such as a cyst or an abscess, but also a primary or metastatic malignant tumor. Although not common, the last two entities should be considered in the evaluation and management of the umbilical masses.

## Competing interests

The authors declare that they have no competing interests.

## Authors' contributions

AL contributed to the conception, design and drafting of the manuscript, as well as to the analysis and interpretation of data. DT contributed to the conception and design of the manuscript and revised it critically. KF contributed to acquisition of data and to the drafting of manuscript. AM contributed to acquisition of data and to the drafting of manuscript. VB contributed to acquisition of data and to the drafting of the manuscript. MT contributed to acquisition of data and to the drafting of the manuscript. SK contributed to the conception and design of the manuscript and revised it critically. All authors read and approved the final manuscript.

## Consent

Written informed consent was obtained from the patient for publication of this case report and accompanying images. A copy of the written consent is available for review by the Editor-in-Chief of this journal.
